# Short-term impact of COVID-19 infection on right ventricular functions: single center observational study

**DOI:** 10.1186/s43044-022-00242-4

**Published:** 2022-02-02

**Authors:** Osama Rifaie, Ahmed Reda, Ahmed Hatata, Amr Gamal, Mostafa Abdelmonaem

**Affiliations:** 1 Department of Cardiology, Faculty of Medicine, Ain Shams University, Cairo, Egypt; 2 Interventional Cardiology Unit, Cardiology Department, Ain Shams University Hospital, Mohamed Hilal Street Villa 10, Abbassia, Cairo, P0 11371 Obour City Egypt

**Keywords:** COVID-19, Right ventricle, Pulmonary artery

## Abstract

**Background:**

COVID-19 pandemic is associated with high morbidity and mortality. Cardiovascular insult is a leading cause of in-hospital mortality in COVID-19 patients, especially right ventricular (RV) dysfunction and massive pulmonary embolism. This study aims to assess short-term impact of COVID 19 infection on (RV) functions among hospitalized patients with moderate or severe illness using bed side trans-thoracic echocardiogram. This study was conducted in 3 isolation hospitals in Cairo, spanning over 3 months during the expected pandemic peak in Egypt in 2020. The study recruited 100 consecutive patients with moderate or severe COVID-19 infection. Four patients refused to participate in the study. Patients with pre-existing structural heart diseases were excluded. All patients underwent full history taking and clinical examination. Bed side echocardiography was done emphasizing on (RV), and (RA) dimensions, (LV) functions and pulmonary artery systolic pressure (PSAS). Cardiac biomarkers were withdrawn and CT angiography was ordered when clinically warranted.

**Results:**

The mean age of the studied cohort was 59.5 ± 8.6 years with males comprising 71.9% of the studied group. (RV) and (RA) dilatation was noted in 8 cases (8.3%). (LV) dysfunction was noted in 11 cases (11.4%). (PASP) showed a statistically significant negative correlation with (LV) function. However, (PSAP) was positively correlated to (RA) and (RV) dimensions, tricuspid regurgitation (TR) jet severity, previous COVID infection and elevated cardiac biomarkers. Mortality was noted in 3 cases (3.1%), all had LV dysfunction with elevated troponin level. Six patients (6.2%) had combined (LV) and (RV) dysfunction.

**Conclusions:**

COVID-19 illness had a negative impact on (RV) and (LV) functions, that could be assessed accurately by trans-thoracic 2 D echocardiogram. The degree of ventricular dysfunction correlated with the rise in cardiac biomarkers as well as the degree of (PASP).

## Background

COVID-19 pandemic, proved to be caused by acute respiratory syndrome—coronavirus 2—(SARS-COV-2), was the direct cause of millions of mortalities and morbidities all over the globe. This viral infection was accused of multi-organ dysfunction, beside a variety of post-survival handicapping sequelae. Acute cardiac complications were the mean reason of in-hospital mortality, including; acute myocardial infarction, life threatening arrythmia, cardiac tamponade, fulminant myocarditis, pulmonary embolism and right ventricular dysfunction [1–4].

A report from Wuhan, China, reported that 12% of 41 patients hospitalized with COVID-19, had signs of cardiovascular involvement as diagnosed by elevated troponin associated with ECG and echocardiographic abnormalities. Since then, other reports have affirmed that cardiac injury can be a part of Corona virus injury [4, 5].

Previous researches focused on biomarkers of myocardial injury as predictors of in-hospital adverse events in COVID-19 patients as high sensitive Troponin and D-dimer. Other preliminary data considered imaging modalities promising tools to predict cardiac events beside its pivotal role in declaring the underling mechanism of cardiovascular complications [6–9].

Provided the clinical relevance of right ventricular involvement in acute respiratory illness and ARDS, 2D trans-thoracic echocardiogram is recommended to evaluate right heart functions among COVID-19 illness as stated by the American society of echocardiography and European Association of cardiovascular imaging (EACVI) [10–14].

Two dimensional echocardiogram provides simple, fast and reliable bed side test to assess cardiac functions and COVID-19 related cardiovascular complications in patients with hemodynamic instability. For issues concerning infection control, routine bed side echo is not recommended for every admitted patient with COVID-19 [15].

## Aim of the work

To assess short-term impact of COVID-19 infection on right ventricular function among hospitalized patients with moderate or severe illness using bed side trans-thoracic echocardiogram.

## Methods

### Study population

Single center observational study conducted in university isolation hospitals. Patients recruitment spanned over 4 months from November 2020 to the end of February 2021 aiming to cover the expected Pandemic peak among Egyptians. One hundred admitted patients were eligible for participation in this study, four patients waived participation, so 96 COVID-19 patients were finally incorporated. A written and informed consent was obtained from every patient. The Study was approved by the Ethic Committee of scientific research.

This study included all admitted patients over 18 years old with moderate or severe COVID-19 infection who accepted to participate in this research. We excluded patients refusing participation and those with pre-existing structural heart diseases.

### Methods

All patients were subjected to the following: A full detailed history including demographic data as age, gender, presence of risk factors as hypertension, diabetes mellitus, dyslipidaemia and smoking. Previous Myocardial infarction and revascularization, rheumatic cardiac affection, arrhythmogenic heart disease, and structural heart disease were also mentioned and considered as exclusion criteria.

All patients underwent a full detailed ECG gated transthoracic echocardiographic study using GE VIVID S 6 apparatus. All operators wore full PPIs. An Echocardiogram was done in left lateral position using apical and parasternal views. The study included detection and quantification of valvular affection (using 2 D, and colour Doppler), evaluation of LV diameters and volumes, segmental wall motion, diastolic function and ejection fraction using M mode and 2D. Assessment of aortic root and left atrial diameters and volume. Evaluation of RV, RA size, RV systolic functions and tricuspid annular plane systolic excursion (TAPSE), right ventricular systolic pressure in mmHg. Evaluation of the pericardium and quantification of pericardial effusion if present. Tissue Doppler assessment of mitral and tricuspid annuli. All echocardiogram reports comply with recommendations of the European society of echocardiography [14]. All saved loops and images were revised by two independent expert interpreters who were blinded about the patient’s clinical data. After each study, the echocardiogram probe was cleaned using hydrogen peroxide disposable wipes. It is worth-mentioning that once echocardiogram was used in COVID-19 isolation zone, it remained in service in this zone and not allowed to be shared by other departments.

### Statistical analysis

Statistical tests were selected and applied according to type of data collected. For qualitative data, frequency and relative frequency, the chi-square was used for analysis. Quantitative variables were expressed as mean ± SD. An independent sample *t*-test analysis was used for the statistical analysis of the continuous data. Univariate then multivariate logistic regression were done to correlate different parameters affecting PASP, Significance was evaluated at *p* < 0.05 levels. The statistical analysis was conducted using the Statistical Package for Social Science (SPSS software version 22).

## Results

### Demographic data and risk factors:

This study included 96 patients with confirmed COVID-19 infection. Mean age of the studied cohort was 59.5 ± 8.63 years with males comprising 71.9% of studied population. 73% of patients were active smokers and only 2% had preexisting chest morbidity (Table 1).

**Table 1:** Demographic data of studied population

	No = 96
*Age (years)*	
Mean ± SD	59.58 ± 8.63
Range	34–77
*Gender*	
Female	27 (28.1%)
Male	69 (71.9%)
*Smokers*	
No	26 (27.1%)
Yes	70 (72.9%)
*Pre-existing chest disease*	
No	94 (97.9%)
Yes	2 (2.1%)

Regarding CT chest on admission, 64 patients had chest findings of CORAD 5, 20 patients had CORAD 4 and 12 had CORAD 3 with a positive PCR test of COVID-19 infection. Four patients were previous survivors of COVID-19. Median CRP was 25 mg/l ranging between 10 and 110 mg/l. All recruited patients had no past history of pre-existing structural heart disease, however during admission, 11 patients showed impaired LV systolic functions (11.3%). Baseline highly sensitive troponin was withdrawn for all subjects, results were positive in 85.4% of patients with moderate to severe illness.

Baseline echocardiographic study revealed; dilated RA dimensions in 8.3% of patients, mean RV base diameter of 36 mm and ranging from 32 to 48 mm, mean TAPSE 19.7 mm ranging from 14 to 28 mm. 7.3% of patients had severe tricuspid incompetence and mean RVSP was 36 mmHg ranging from 20 to 65 mmHg (Table 2). CT pulmonary angiography was ordered in patients highly suspected of pulmonary emboli and 10 patients were diagnosed during hospital stay with pulmonary embolism, four of them necessitated thrombolytic therapy.

**Table 2:** Baseline investigation and laboratory work up on admission

	No = 96
*CT CHEST*	
CORAD 3	12 (12.5%)
CORAD 4	20 (20.8%)
CORAD 5	64 (66.7%)
*CT pulmonary angiogram proven pulmonary embolism*	
No	86 (89.6%)
Yes	10 (10.4%)
*TLC /cmm*	
Mean ± SD	16.90 ± 3.76
Range	11–30
*Serum ferritin*	
High	96 (100.0%)
*CRP mg/l*	
Median(IQR)	25 (20.5–32.5)
Range	10–110
*ESR mm 1st h*	
Median(IQR)	32 (23–43.5)
Range	10–102
*RV Base dimensions mm*	
Mean ± SD	36.44 ± 3.18
Range	32–48
*RA*	
Normal	88 (91.7%)
Dilated	8 (8.3%)
*TAPSE mm*	
Mean ± SD	19.71 ± 2.35
Range	14–28
*TR JET*	
Moderate	59 (61.5%)
Mild	30 (31.2%)
Severe	7 (7.3%)
*RVSP (mmHg)*	
Mean ± SD	36.34 ± 8.62
Range	20–65
*RVSP groups*	
Normal RVSP	26 (27.1%)
High RVSP (≥ 35mmhg)	70 (72.9%)
*PREVIOUS COVID INEFCTION*	
No	92 (95.8%)
Yes	4 (4.2%)
*Mortality*	
No	93 (96.9%)
Yes	3 (3.1%)
*Troponin and CK*	
Positive	82 (85.4%)
Negative	14 (14.6%)
*LV systolic function*	
Normal (EF ≥ 50%)	85 (88.5%)
Impaired	11 (11.5%)

Regarding hospital course, 93 patients were discharged to home in fair condition and unfortunately, 3 patients died during hospital stay from COVID-19 systemic complications.

### Factors influencing pulmonary artery pressure (Table 3)

**Table 3:** Factors influencing pulmonary artery pressure

	RVSP (mmHg)		Test value	*p*-value	Sig
	Mean ± SD	Range			
*SEX*					
Female	36.19 ± 9.63	25–65	0.255•	0.799	NS
Male	36.41 ± 8.26	20–55			
*Smoker*					
No	34.85 ± 7.38	25–60	1.038•	0.302	NS
Yes	36.9 ± 9.02	20–65			
*HISTORY OF CHEST PRB*					
No	36.48 ± 8.63	20–65	1.053•	0.295	NS
Yes	30 ± 7.07	25–35			
*CT CHEST*					
CORAD 3	38.17 ± 7.4	30–55	1.064^‡^	0.349	NS
CORAD 4	38.15 ± 10.03	28–65			
CORAD 5	35.44 ± 8.34	20–60			
*CT PULMONARY ANGIO*					
No	35.9 ± 8.13	20–65	1.505•	0.136	NS
Yes	40.2 ± 11.86	25–55			
*RA*					
Normal	34.66 ± 6.64	20–55	8.335•	**0.000**	**HS**
Dilated	54.88 ± 5.54	48–65			
*TR JET*					
Moderate	37.81 ± 6.47	20–55	64.081	**0.000**	**HS**
Mild	29.13 ± 3.33	20–38			
Severe	54.86 ± 5.98	48–65			
*PREVIOUS COVID INEFCTION*					
No	35.92 ± 8.51	20–65	2.343•	**0.021**	**S**
Yes	46 ± 4.83	42–53			
*Mortality*					
No	36.28 ± 8.61	20–65	0.405•	0.687	NS
Yes	38.33 ± 10.41	30–50			
*Troponin and CK*					
Not elevated	34.5 ± 6.8	20–55	5.912•	**0.000**	**HS**
Yes	47.14 ± 10.34	30–65			
*LV systolic function*					
Normal	34.71 ± 6.9	20–55	6.079•	**0.000**	**HS**
Impaired	49 ± 10.32	30–65			

Bold indicates highly significant

•: Independent t-test; ‡: One Way ANOVA test

Gender difference and age did not show any statistical relation to pulmonary artery pressure, smokers had numerically higher pulmonary artery pressure with no statistical significance. The severity of right atrial dimensions and jet of tricuspid incompetence were statistically correlated to pulmonary systolic pressure. Survivors of previous COVID-19 infections had statistically relevant higher PASP as compared to other patients with de novo COVID-19 infection. Troponin and creatinine kinase showed a highly significant relationship with PASP with *p* value of 0.0001. De novo decline in LV systolic functions showed a high statistical relation to PASP with *p* value of 0.0001.

**Fig. 1:**
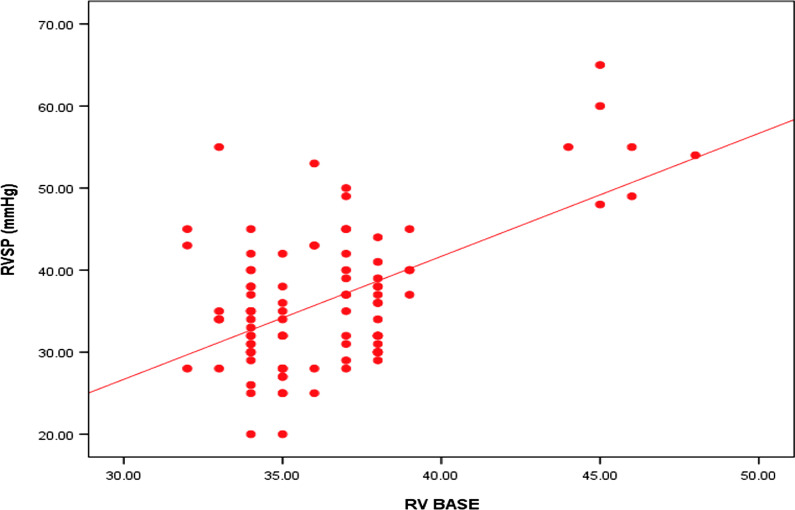
Spearman correlation between RVSP and RV base diameter

Table 4 shows the results of univariate analysis to correlate different parameters affecting PASP, the only significant predictor was right ventricular basal diameter (Fig. 1).

**Table 4:** Logistic regression analysis of factors influencing RVSP,

	RVSP (mmHg)	
	*r*	*p*-value
AGE	− 0.095	0.355
TLC (thousands)	0.199	0.052
CRP	0.025	0.809
ESR	− 0.041	0.693
RV BASE	0.309**	**0.002**
TAPSE	− 0.043	0.680

Bold indicates highly significant

*r* = Spearman correlation coefficients ** = Significant *p* value

Patients were divided to 2 groups according to RVSP; group with normal RVSP (26 patients) and another group with high RVSP exceeding 35 mmHg (70 patients). Severity of TR jet and right ventricular basal dimensions showed statistically significant relation to group with high RVSP with *p* value of 0.00 and 0.04, respectively (Table 5).

**Table 5:** Comparison between patients with normal and high RVSP

	Normal RVSP	High RVSP	Test value	*p*-value	Sig
	(31.2 ± 3.1)	(45 ± 10.3)			
	No. = 26	No. = 70			
*AGE*					
Mean ± SD	61.12 ± 9.95	59.01 ± 8.09	1.061•	0.291	NS
Range	34–76	38–77			
*SEX*					
Female	8 (30.8%)	19 (27.1%)	0.123*	0.725	NS
Male	18 (69.2%)	51 (72.9%)			
*SMOEKR*					
No	7 (26.9%)	19 (27.1%)	0.000*	0.983	NS
Yes	19 (73.1%)	51 (72.9%)			
*HISTORY OF CHEST PRB*					
No	25 (96.2%)	69 (98.6%)	0.543*	0.461	NS
Yes	1 (3.8%)	1 (1.4%)			
*CT CHEST*					
CORAD 3	2 (7.7%)	10 (14.3%)	0.923*	0.630	NS
CORAD 4	5 (19.2%)	15 (21.4%)			
CORAD 5	19 (73.1%)	45 (64.3%)			
*CT PULMONARY ANGIO*					
No	22 (84.6%)	64 (91.4%)	0.943*	0.331	NS
Yes	4 (15.4%)	6 (8.6%)			
*RA*					
Normal	26 (100.0%)	62 (88.6%)	3.242*	0.072	NS
Dilated	0 (0.0%)	8 (11.4%)			
*TR JET*					
Moderate	5 (19.2%)	54 (77.1%)	40.925*	**0.000**	**HS**
Mild	21 (80.8%)	9 (12.9%)			
Severe	0 (0.0%)	7 (10.0%)			
*TLC (thousands)*					
Mean ± SD	15.90 ± 2.64	17.27 ± 4.05	− 1.607•	0.111	NS
Range	11–20	11–30			
*CRP*					
Median (IQR)	28.5 (22–45)	24.5 (20–31)	0.929^‡^	0.353	NS
Range	10–110	10–110			
*ESR*					
Median (IQR)	30.5 (24–40)	32 (22–44)	0.012^‡^	0.990	NS
Range	18–102	10–100			
*RV BASE*					
Mean ± SD	35.35 ± 1.70	36.84 ± 3.50	− 2.086•	**0.040**	**S**
Range	32–38	32–48			
*TAPSE*					
Mean ± SD	19.69 ± 1.85	19.71 ± 2.52	− 0.041•	0.968	NS
Range	17–23	14–28			
*PREVIOUS COVID INEFCTION*					
No	26 (100.0%)	66 (94.3%)	1.550*	0.213	NS
Yes	0 (0.0%)	4 (5.7%)			
*Mortality*					
No	25 (96.2%)	68 (97.1%)	0.061*	0.805	NS
Yes	1 (3.8%)	2 (2.9%)			
*Troponin and CK*					
Not elevated	25 (96.2%)	57 (81.4%)	3.300*	0.069	NS
Yes	1 (3.8%)	13 (18.6%)			
*LV systolic function*					
Normal	25 (96.2%)	60 (85.7%)	2.037*	0.154	NS
Impaired	1 (3.8%)	10 (14.3%)			

Bold indicates highly significant

*: Chi-square test; •: Independent t-test; ‡: Mann-Whitney test

## Discussion

It is understood that severe COVID-19 infection causes multi-organ dysfunction, and cardiac injury yields worse outcomes [16]. Trans-thoracic echocardiography is helpful in this group of patients but not routinely ordered to minimize the risk of disease transmission [17]. It enables bed side and non-invasive assessment of heart function and hemodynamic status of the patient [16]. Conventional echocardiography study can rule out obstructive problems (e.g., cardiac tamponade and pulmonary embolism) and hypovolemic shock (decreased cardiac output and collapsed IVC) [17–20]. In recent years, many studies observed that nearly 40% of severe sepsis patients developed a decline in left ventricular systolic and diastolic functions. In severe sepsis hyperdynamic status increases cardiac performance due to systemic inflammatory response. In later stages, hypoxia and inflammation cause myocardial suppression [20].

The main finding of this study was that COVID-19 negatively affects the RV as well as LV functions. Moreover, we found a strong correlation between right ventricular systolic pressure and elevated cardiac biomarkers, right ventricular dimensions as well as with newly recognized left ventricular systolic dysfunction. This finding confirmed the injurious effect of COVID-19 infection on myocardial performance. COVID-19 myocardial impact is not only on pulmonary artery pressure and right ventricular dimensions but also it harms left ventricular systolic functions which were directly correlated to high recording of right ventricular systolic pressure. The magnitude of rise of cardiac biomarkers was correlated to high right ventricular systolic pressure assessed by echocardiography, supporting the hypothesis of using either cardiac biomarkers or bed side imaging to predict right ventricular injury and consequently predicting worse outcome during hospital stay. In fact, the 3 patients who died in this study had LV dysfunction and elevated Troponin serum level. Patients with previous COVID-19 infection who were re-admitted by moderate or severe infection were more liable for de novo myocardial injury in terms of elevated pulmonary artery pressure.

These findings were in accordance with Barman et al. who showed a statistically positive correlation between RV diameter and troponin level, similarly left ventricular systolic functions showed a statistically significant negative correlation with troponin and RV systolic functions. In their linear regression model; Troponin, left ventricular ejection fraction, D-dimer, RA dimensions and PASP were independent predictors of RV dilatation [21].

Further evidence of RV involvement in COVID-19 was mentioned by D’Andrea et al., who reported a statistically significant association between RV dilation/systolic dysfunction and mortality in a cohort of 115 patients. Patients with RV dysfunction had signs of myocardial injury (elevated Troponin), more severe lung disease, and higher pulmonary artery pressure, suggesting increased afterload as the primary mechanism of RV dysfunction in patients with severe COVID-19 sepsis [22]. In line with these findings, Kim et al. released data from 510 patients who underwent echocardiography in three NY hospitals from March to May 2020 [20]. RV dilation was detected in 35% of the cohort, while RV systolic dysfunction was found in 15%. These changes were associated with higher levels of circulating biomarkers. RV abnormalities were associated with lower LVEF, and associated with higher pulmonary artery systolic pressure, supporting an increased pulmonary vascular load as the primary pathophysiological mechanism. In multivariate analysis, RV abnormalities independently predicted mortality (32% of the cohort) [23].

In this study, TAPSE as a marker of RV systolic functions did not show a statistically relevant correlation to PASP, despite the strong correlation between RV basal dimensions and PASP. This finding could be attributed to the diversity of factors influencing TAPSE measurements as LV systolic functions, ischemic etiology of heart failure and wall motion index of RV [24]. Another point, is the impact of TR jet severity on reproducibility of TAPSE, severe TR is a confounding factor when TAPSE is used to assess RV systolic functions [25].

Despite the crucial rule of bed side echocardiogram in evaluating RT ventricular functions and its impact in predicting patient course during his hospital stay, the decision to perform echocardiogram should be only when clinically warranted and with the use of full personal protective equipments to minimize virus transmission. Moreover, the need for follow up study for a COVID-19 patient should be based upon multidisciplinary treating team since the effect of COVID-19 on ventricular function might be extended to longer periods.

## Conclusions

COVID-19 illness had a negative impact on right ventricular as well as left ventricular functions, which could be assessed accurately by trans-thoracic 2 D echocardiogram. RV dimensions increased with pulmonary artery systolic pressure. The latter matched with the rise in cardiac biomarkers.

## Limitation

This was a single center study with a relatively small sample size. We are in need of multi-center prospective study with long-term follow up to understand COVID-19 illness myocardial injury and propose solutions to minimize patient morbidity and mortality.

## Data Availability

All Data including angiogram films and stored echocardiographic loops are available with the authors and in Ain Shams University echocardiography records.

## Abbreviations

RV: Right ventricle
LV: Left ventricle
RVSP: Right ventricular systolic pressure
LVEF: Left ventricular ejection fraction
TR: Tricuspid regurgitation
TAPSE: Tricuspid annular plane systolic excursion
PASP: Pulmonary artery systolic pressure
2D: Two dimensional
